# Justification for the use of *Ocimum gratissimum *L in herbal medicine and its interaction with disc antibiotics

**DOI:** 10.1186/1472-6882-9-37

**Published:** 2009-09-28

**Authors:** Emeka I Nweze, Elizabeth E Eze

**Affiliations:** 1 Department of Microbiology, University of Nigeria, Nsukka, Enugu State, Nigeria; 2 Department of Dermatology, Case Western Reserve University, Cleveland, Ohio, USA

## Abstract

**Background:**

The ethanolic extract of the leaves of *Ocimium gratisimum *L. (Lamiaceae), used in traditional medicine for the treatment of several ailments such as urinary tract, wound, skin and gastrointestinal infections, was evaluated for its antibacterial properties against four clinical bacteria isolates namely: *Escherichia coli, Proteus mirabilis, Staphylococcus aureus and Pseudomonas aeruginosa *and the antifungal properties using a clinical isolate of *Candida albicans*. A typed bacterium of *Escherichia coli *ATCC 11775 and another typed fungal strain of *Candida albicans *(ATCC 90028) were also included. The study also intended to verify if the concomitant administration of conventional antibiotics with *Ocimium gratisimum *which is normally taken as food (spice) will negatively affect its activity.

**Methods:**

The agar diffusion method was used to test the in vitro activity of the plant extract. The interaction of the plant extract with some disc antibiotics namely: ciprofloxacin, septrin, streptomycin, ampicillin, nystatin and ketoconazole was tested using the agar overlay inoculum susceptibility disc method. Phytochemical analysis of the extract was performed following established methods.

**Results:**

The extract showed good but varying *in vitro *activities against all the isolates tested. While ampicillin showed synergistic interaction with the plant extract against clinical isolates of *E. coli *and *P. mirabilis*, septrin was synergistic against the clinical isolate of *E. coli *only. Similarly, the activity of the extract against *C. albicans *isolate was synergistic with ketoconazole and nystatin.

**Conclusion:**

The study has validated the folkloric use of *O. gratissimum *in traditional medicinal practice and goes further to show that the use of this plant material as food spice may not really threaten the efficacy of some conventional antibiotics that may have been taken concomitantly with it as is the popular belief in the practice of herbal medicine in local/rural communities of many countries in the world.

## Background

The use of plant materials as spices, condiments and for medicinal purposes dates back to the history of mankind [1-3]. Recently, the exploitation of wild plants for medicinal purposes has gained more acceptances in many countries of the world. To further underscore the importance of herbal medicine, most national governments have established the traditional medicine regulatory council under the supervision of their various health ministries to tap the numerous potentials of herbs. This may be because traditional medicine has long been practiced even before the orthodox medical practice appeared [4]. *Ocimum gratissimum *belongs to the group of plants known as spices. The plant is an erect small plumb with many barnacles usually not more than 1 m high [5]. It is of the family Labiatea, genus *Ocimum *and species *gratissimum *[6]. The plant is found throughout the tropics and subtropics and its greatest variability occurs in tropical Africa and India [7]. In South East Asia, it is cultivated as a home garden crop but it is grown on a commercial scale in Vietnam. It is used for a variety of reasons. In culinary, it is used in salads, soups, pastas, vinegars and jellies in many parts of the world. The Thai people are popularly known to use it in food flavouring. In traditional medicine, the leaves have been used as a general tonic and anti-diarrhea agent and for the treatment of conjunctivitis by instilling directly into the eyes; the leaf oil when mixed with alcohol is applied as a lotion for skin infections, and taken internally for bronchitis. The dried leaves are snuffed to alleviate headaches and fever among other uses [6]. Although, conventional antibiotics have been very useful in orthodox medicine, it has been argued by many that its concomitant use with herbal extracts is not desirable as one normally antagonizes the activity of the other. Considering the fact that *Ocimum gratissimum *is used in most local dishes/foods to achieve a variety of purposes, there is need to ascertain if its extract antagonizes or acts as a synergy when used together with conventional antibiotics. In addition, despite the fact that the various extracts of *O. gratissum *have been tested *in vitro *and shown to be active against some bacteria and fungal isolates [8-11], specific strain differences supposes that a lot more strains of bacteria and fungi across other regions be tested to ascertain their *in vitro *activity against this spice and this was one of the motivations for our study. This study thus investigated the antibacterial and antifungal properties of *O. gratissimum *ethanolic leaf extract and its interaction with some disc antibiotics.

## Methods

### Identification and preparation of plant material

The plant, *Ocimum gratissimum *was purchased from Ogige market in Nsukka, Enugu state, Nigeria. The plant was identified by A.O Ozioko, a former plant taxonomist at the herbarium section of the Department of Botany, University of Nigeria, Nsukka, Nigeria. Voucher specimens were deposited accordingly. The leaves of the plant were first separated from the stalk, rinsed with water to remove dirt; air dried at room temperature and ground to fine powder using an electric blender as previously described [2]. Extraction was performed by adding a 100 g of the ground leaf powder in 900 ml of ethanol in a sterile flask, swirled to ensure effective mixing and stoppered to avoid loss of volatile liquid at ambient temperature (28 ± 2°C). The mixture was extracted by agitation on a rotary shaker. After 48 hrs, the mixture was decanted. The filtrate was then poured into stainless trays and the extract was allowed to evaporate to dryness at room temperature (28 ± 2°C) for 2-3 days under the microbiological hood set at appropriate conditions or by using a rotary evaporator in order to concentrate it. The concentrated extract was then scooped into a pre-weighed small sterile container and weighed. The difference between the two weights was recorded as the percentage yield. The container was then covered, labeled accordingly and stored in the refrigerator and was later reconstituted as described previously [2,12] and used for the tests. The extract yield was 8.5%.

### Test isolates

Resistant clinical isolates of *P. aeruginosa, P. mirabilis, Staphylococcus aureus*, *E. coli, C. albicans *and two control strains *E. coli *ATCC 11775 and *C. albicans *ATCC 90028 were *used *in this study.

### Preparation and standardization of inoculum

All isolates were first subcultured to obtain fresh cultures. The inoculum of each isolate was then prepared according to the NCCLS approved guideline for bacteria and yeasts [13] as previously described [2].

### Antibiotic Sensitivity discs

Gram negative sensitivity discs manufactured by Potum laboratories (India) were purchased and used for the study. The disc contained 10 μg of ciprofloxacin (CPX), 30 10 μg of streptomycin (S), 30 μg of septrin (SXT) and 30 μg of ampicillin (PN). For the antifungal antibiotics discs of ketoconazole (KET) and nystatin (NYS), Whatmann No 1 filter paper was cut to give 6 mm diameter paper discs. The paper discs were sterilized in hot air oven and later soaked in the prepared desired concentration of the two antifungal antibiotics used in the study and then allowed to dry and used for the tests.

### Phytochemical analysis of extract

The methods described by Harborne [14] with minor modifications as described previously [2] were used to test for the presence of the active ingredients in the test sample. The phytochemicals tested were tannins, alkaloids, flavonoids, terpenes, saponins, carbohydrates and cyanogenetic glycosides.

### Determination of the antimicrobial activity of the extract

One gram of the extract was measured into a sterile test tube and 10 ml of 20% dimethyl sulfur oxide (DMSO) added to dissolve it. This gave a 100 mg/ml concentration of the extract and this was diluted in two-folds to obtain four different dilutions of the extract: 50 mg/ml,25 mg/ml,12.5 mg/ml and 6.25 mg/ml in addition to the 100 mg/ml concentration. Using a sterile cork borer of diameter 8 mm, 5 wells were dug into the Mueller Hinton agar medium which was initially flooded with 1 ml of the standardized inoculum of each test organism and the excess was drained using a Finn pipette. Each well was then filled with about 100 μl of the different concentrations of the crude extract. The plates were left for one hour at room temperature for diffusion to take place and later incubated for 18-24 hrs at 37°C. The experiment was performed in triplicates and the inhibition zone diameter was recorded as the average of the three replicates.

### Determination of the interaction between the plant extract and conventional antibiotics

A solution of the crude extract was diluted in Mueller Hinton agar to concentrations of 25 mg/ml and 6.25 mg/ml. Twenty five mg/ml was obtained as the sub-inhibitory concentration for *P. aeruginosa, S. aureus *and typed strain and clinical isolates of *C. albicans *while 6.25 mg/ml was the sub-inhibitory concentration for *P. mirabilis *and *E. coli *(typed and clinical). Twenty ml of the extract agar mixture were placed in a Petri dish to form the base extract layer; 2 ml of the molten agar containing 0.2 ml of the test isolate was applied as a thin overlay inoculum agar layer and allowed to solidify. Antibiotic discs were then placed on the solidified surface. This was done for each of the organism. For the control, 20 ml of molten agar was poured on a second Petri dish to produce an extract-free agar layer. The overlay inoculum agar layer was prepared as described above. Antibiotic discs were placed ascetically on the solid surface and this plate was taken as the control. This was done for each organism. Plates from the combination and the control were incubated at 37°C for 18 - 24 hours. The experiment was performed in triplicates and the inhibition zone diameter (IZD) was measured separately and the average of the three replicates were recorded.

## Results

The result of the phytochemical analysis of the *O. gratissimum *plant extract is shown in Table 1. The extract contained fats and oil in higher quantities (+++), followed next by reducing sugars and the terpenes (++). Cyanogenetic glycosides, saponins, alkaloids and steroidal aglycone were next (+) while tannins, anthroquinone and flavonoid were entirely absent. Table 2 shows the inhibition zone diameters by the test isolates against different concentrations of the plant extract. Concentrations of 12.5 and 25 mg/ml inhibited only *P. mirabilis*. However, concentrations of 100 and 50 mg/ml showed varying inhibition zone diameters on all the isolates tested in the study. Typed and clinical isolates of *E. coli *could not be inhibited at any of the concentrations. Table 3 shows the inhibition zone diameters shown by the conventional antibiotics on the bacterial and the yeast isolates. Like expected, there were varying inhibition of all the isolates used in the study. It also presents the results of the in vitro interaction studies of the plant extract and the various antibiotics and summarizes the result of the interaction of the plant extract and the various antibiotics. Out of the twenty four different combinations, nine (37.5%) were synergistic, three (12.5%) were additive, two (8.3%) were indifferent, seven (29.1%) were antagonistic while three (122.5%) showed no activity. In general, it implies that only 29.1% of all the combinations were antagonistic as compared to 70.9% which were not. The chi square test was used to calculate the statistical significance and it was found to be statistically significant (P < 0.05). The minimum inhibitory concentrations (MICs) which are the least concentrations of the plant extract that inhibited bacterial growth were 50 mg/ml for *S. aureus, P. aeruginosa, C. albicans *(ATCC 90028), *C. albicans *and 12.5 mg/ml for *P. mirabilis*.

**Table 1:** Results of the phytochemical analysis of *Ocimium gratissimum *leaf extract

**Test substances**	**Concentrations**
Tannins	+
Cyanogenic glycosides	+
Anthroquinones	-
Saponins	+
Terpenes	++
Flavonoids	-
Alkaloids	+
Reducing sugar	++
Fats and oil	+++
Steroidal aglycone	+

Key: - Not present; +, present at low concentration; ++, present at moderate concentration; +++, present at high concentration

**Table 2:** The inhibition zone diameters shown by the test isolates against different concentrations of the plant extract

**Test isolates**	**Diameter shown by different concentrations of the extract (mm)**				
**Clinical/typed strains**	**100 mg/ml**	**50 mg/ml**	**25 mg/ml**	**12.5 mg/ml**	**6.25 mg/ml**

*S. auerus*	19	13.5	-	-	-
*P. aeruginosa*	11	9.8	-	-	-
*P. mirabilis*	22	21	20	11	-
*E. coli ATCC 11775*	-	-	-	-	-
*E. coli*	-	-	-	-	-
*C. albicans ATCC 90028*	13	10	-	-	-
*C. albicans*	16	13	-	-	-
DMSO	-	-	-	-	-

Key: -, No inhibition. The values are average figures of three replicates.

**Table 3:** Summary of the interaction of the plant extract and the various antibiotics

**Test isolates**	**Antibiotic**	**Antibiotic alone (mm)**	**Antibiotic plus plant extract (mm)**	**% change in IZD**	**Interpretation**
*S. aureus*	CP	16	16	0	indifference
	SX	15	15	0	indifference
	PN	-	-	-	-
	SE	24	22	-8.33	antagonism
					
*P. aeruginosa*	CP	29	35	20.69	synergism
	SX	23	12	-47.83	antagonism
	PN	-	-	-	-
	SE	29	-	-	-
					
*P. mirabilis*	CP	15	25	66.67	synergism
	SX	24	26	8.33	additive
	PN	12	20	66.67	synergism
	SE	29	19	-34.48	antagonism
					
*E. coli*	CP	35	42	20	synergism
*ATCC 11775*	SX	10	12	20	synergism
	PN	16	10	-37.5	antagonism
	SE	22	11	-50	antagonism
					
*E. coli*	CP	39	40	2.56	additive
	SX	10	12	20	synergism
	PN	8	10	25	synergism
	SE	14	11	-21.42	antagonism
					
*C. albicans*	*KE*	26	23	-11.54	antagonism
					
*ATCC 90028*	*NY*	38	30	-7.14	antagonism
					
*C. albicans*	KE	21	27	28.57	synergism
	NY	24	32	33.33	synergism

Key: -, No activity; CP, 10 mg of ciprofloxacin; SX, 30 mg of septrin; SE, 30 mg of streptomycin; PN, 30 mg of ampicillin; KE, ketoconazole; NY, nystatin.; %, percentage; IZD, inhibition zone diameter.

## Discussion

In agar diffusion assays, the inhibitory zone diameter produced is the result of the growth of the test organisms and the diffusion of the test agents through the agar, both events occurring simultaneously [15] so it then means that any factor which affects the rate of microbial growth or rate of diffusion of the suspected antimicrobial agent under test, will invariably affect the result. The factors which were pertinent to the formation of inhibition zone included the type/nature of the test organisms, the size of inoculum, the culture media (which should be able to support the growth of organisms and not interfere with diffusion or activity of test organisms) and the temperature of incubation [16]. Secondary metabolites of plants such as saponins, flavonoids, tannins, carbohydrates, cyanogenetic glycosides, reducing sugar and all other active principles of plants have been shown to be responsible for the antimicrobial activities shown by these extracts. [17,2]. However from the phytochemical analysis of this plant extract, some of these secondary metabolites were absent (tannins, anthroquinone and flavonoid), some in low and moderate concentrations and a few in high concentration. However, the antimicrobial activities shown by the extract will likely be due to one or more of the several other phytochemical constituents shown to be present in the extract (Table 1). There was no activity against *E. coli *for both the typed culture and the clinical isolate, as opposed to information in literature [8]. This may be due to the absence of some secondary metabolites or the presence of some in low concentration; or it may be due to the type of strains used or a slight change in any of the factors mentioned earlier that are likely to affect rate of microbial growth or rate of diffusion of the test agent. A percentage change in the inhibition zone diameter was used to draw inference on the effects of the interaction on the test organisms. It has been suggested that for a synergistic effect, the diameter of the zone of inhibition in the test plate (plate containing the plant extract and the antibiotics) should be greater than that in the control plate (plate containing extract-free base agar layer) by at least 19%. A percentage increase in the inhibition zone diameter that is lower than 19% indicates additivity. Where the inhibition zone diameters in the test and the control are equal, the combined antibiotics have indifferent effect and if the zone diameter in the test is less than the one in the control, then there is antagonism [18]. It was also observed that for *S. aureus *it was only ampicillin that did not show activity against it. This may be due to the fact that *S. aureus *usually develops resistance to β-lactam drugs. There was antagonism in inhibitory zone diameter when streptomycin was combined with the extract. Septrin and ciprofloxacin showed indifferent effects individually when they were combined with the extract. For *P. mirabilis*, all the test antibiotics showed activity. However when combined with the plant extract, it was observed that streptomycin showed antagonism. Ciprofloxacin and ampicillin were synergistic when each was combined with the extract. However with septrin, there was additivity. Against *P. aeruginosa *all test antibiotics showed activity, but on combining with the plant extract, ciprofloxacin showed synergism while septrin was antagonistic. Ampicillin and streptomycin did not have any individual activity when combined with the extract. The antibiotics were all active against the clinical isolate of *E. coli *and the typed culture, when the extract was combined with either ciprofloxacin or septrin, synergistic interaction was observed. Moreover, ampicillin and septrin when combined together with either ciprofloxacin or septrin showed synergistic interaction. Moreover, ampicilin and streptomycin when combined separately with the extract showed antagonism against *E. coli *(ATCC 11755). Against the clinical isolate of *E. coli *ciprofloxacin was additive, streptomycin was antagonistic while septrin and ampicillin were synergistic. Nakamura *et al *[8] found that the essential oil of *O. gratissimum *has antibacterial activity against *Shigella flexineri, E. coli, Klebsiella *spp and *Proteus mirabilis*. Ketoconazole and nystatin were both active against the clinical isolate of *C. albicans *and the type culture. The effect of interaction between the plant extract and the antibiotics was synergistic on *C. albicans*. Against *C. albicans *(ATCC 90028), ketoconazole showed antagonism while nystatin was additive. In a previous study, using agar dilution technique, Silva *et al *[10] demonstrated that *O. gratissimum *exhibited antifungal activities against the dermatophytes: *M. canis, M. gypseum, T. rubrum and T. mentagrophytes. *Lemos and co-workers [11] also showed that the ethanolic crude extract and the ethyl acetate, hexane, chloroform, essential oil and eugenol of *O. gratissimum *have antifungal activities against *C. neoformans. *Generally, it should be remembered that the study is in vitro, and the likelihood of possibility of change in activity of microflora of a patient cannot be ruled out as it functions in vivo. Also, the possibility of alteration of the presence of other exogenous compound and the significance of this alteration in modifying drug interaction will only be determined by detailed pharmacokinetic studies that relate to the distribution of drugs metabolized by the microflora and their potentially active metabolites to the therapeutic response of the patient to the antibiotic.

Conclusively, the result of the interaction of the leaves of the plant with the antibiotics investigated in this study was thought to be basically due to the effects of the phytochemical constituents of the plants. Ciprofloxacin showed the most synergistic effect on the bacterial isolates, followed by septrin and ampicillin. Streptomycin was antagonistic while ketoconazole and nystatin were synergistic on only the clinical isolate of *C. albicans*. The antibacterial activity shown by the leaf extract *O. gratissimum *corresponds with the traditional medicinal use of the plant in some parts of the world [9]. The plant is known to be used in the treatment of wound infections for which most of the bacterial species investigated in our study are known to be the etiological agents.

## Conclusion

The study has therefore proven that *O. gratissimum *has some antimicrobial effect and can be further investigated for possible used in formulation of antimicrobial compounds.

## Competing interests

The authors declare that they have no competing interests.

## Authors' contributions

EIN conceived the study, participated in its design and coordination, provided logistics, repeated the phytochemical assay and wrote the manuscript. EEE did the antimicrobial assay and the initial phytochemical analysis and obtained the plant materials. All authors read and approved the final manuscript.

## Pre-publication history

The pre-publication history for this paper can be accessed here:



## References

[B1] Garland S (1972). The Herbs and Spices book.

[B2] Nweze EI, Okafor JI, Njoku O (2004). Antimicrobial activities of methanolic extracts of *Trema guineensis *(Schumm and Thorn) and *Morinda Lucida *Benth used in Nigeria herbal medicinal practice. Journal of Biological Research and Biotechnology.

[B3] Ogunyemi MA (1979). The origin of the herbal cure and its spread.

[B4] Okafor JI, Nweze EI, Njoku OU (2001). Antimicrobial activities of the methanolic extracts of *Zapotica portericensis *Benth and *Cissus quadrangularis *Linn. Nigeria Journal of Pure and Applied Sc.

[B5] Vierra RF, Simon JE (2000). Chemical characterization of Ocimum gratissimum found in the market and used in Traditional medicine in Brazil. Journal of Economic Botany.

[B6] Iwu MM (1993). Handbook of African medicinal plants.

[B7] Aruna K, Sivaramakrishina VM (1990). Plants as protective agents against cancer. Indian Journal of Experimental Biology.

[B8] Nakamura CV, Ishid K, Faccin LC, Filho BP, Cortez DA, Rozental S, Souza W, Ueda N (2004). *In vitro *activities of essential oils from *Ocimum gratissimum *against four *Candida *species. Research in Microbiology.

[B9] Nakamura CV, Nakamura T, Bando E, Melo AFN, Cortez DAG, Filho BPD (1999). Antibacterial activities of *Ocimum gratissimum *L essential oil. Mem Inst Oswaldo Cruz.

[B10] Silva MRM, Oliveira JG, Fernades OFL, Passos XS, Costa CR, Souza LKH, Lemos JA, Paula JR (2005). Antifungal activities of *Ocimum gratisimum *towards dermatophytes. J Ethnopharmacol.

[B11] Lemos JA, Passos XS, Fernandes OF, Paul JR, Ferri JP, Ferri PH, Souza LK, Lemos AA, Silva KRM (2005). Antifungal activity of *Ocimum grattisimum *towards *Cryptococcus neoformans*. Journal of Ethnopharmacology.

[B12] Nweze EI, Okafor JI, Njoku OU (2005). Antifungal activities of pair combinations of extracts from *Morinda lucida *Benth by Decimal additive assay. Journal of Biological Research and Biotechnology.

[B13] National Committee for Clinical Laboratory Standards (2002). Reference method for broth dilution susceptibility testing of bacteria and yeasts: Approved standard. NCCLS document M35-A.

[B14] Harbone JB (1973). Phytochemical methods: A guide to modern techniques of plant analysis.

[B15] Trease EG, Evans CW (1978). Laboratory Methods in Antimicrobial Chemotherapy.

[B16] Nester EW, Anderson DG, Roberts CE, Pearsal NN, Nester MI (2004). Microbiology: A human perspective.

[B17] Cowan MM (1999). Plant products as antimicrobial agents. Clinical Microbiology Reviews.

[B18] Okore CV (2005). Pharmaceutical Microbiology.

